# Describing the performance of U.S. hospitals by applying big data analytics

**DOI:** 10.1371/journal.pone.0179603

**Published:** 2017-06-29

**Authors:** Nicholas S. Downing, Alexander Cloninger, Arjun K. Venkatesh, Angela Hsieh, Elizabeth E. Drye, Ronald R. Coifman, Harlan M. Krumholz

**Affiliations:** 1 Center for Outcomes Research and Evaluation, Yale-New Haven Health, New Haven, Connecticut, United States of America; 2 Department of Mathematics, Yale University, New Haven, Connecticut, United States of America; 3 Department of Emergency Medicine, Yale School of Medicine, New Haven, Connecticut, United States of America; 4 Department of General Pediatrics, Yale School of Medicine, New Haven, Connecticut, United States of America; 5 Department of Internal Medicine, Section of Cardiovascular Medicine, Yale School of Medicine, New Haven, Connecticut, United States of America; 6 Department of Internal Medicine, Robert Wood Johnson Foundation Clinical Scholars Program, Yale School of Medicine, New Haven, Connecticut, United States of America; 7 Department of Health Policy and Management, Yale School of Public Health, New Haven, Connecticut, United States of America; Stanford University School of Medicine, UNITED STATES

## Abstract

Public reporting of measures of hospital performance is an important component of quality improvement efforts in many countries. However, it can be challenging to provide an overall characterization of hospital performance because there are many measures of quality. In the United States, the Centers for Medicare and Medicaid Services reports over 100 measures that describe various domains of hospital quality, such as outcomes, the patient experience and whether established processes of care are followed. Although individual quality measures provide important insight, it is challenging to understand hospital performance as characterized by multiple quality measures. Accordingly, we developed a novel approach for characterizing hospital performance that highlights the similarities and differences between hospitals and identifies common patterns of hospital performance. Specifically, we built a semi-supervised machine learning algorithm and applied it to the publicly-available quality measures for 1,614 U.S. hospitals to graphically and quantitatively characterize hospital performance. In the resulting visualization, the varying density of hospitals demonstrates that there are key clusters of hospitals that share specific performance profiles, while there are other performance profiles that are rare. Several popular hospital rating systems aggregate some of the quality measures included in our study to produce a composite score; however, hospitals that were top-ranked by such systems were scattered across our visualization, indicating that these top-ranked hospitals actually excel in many different ways. Our application of a novel graph analytics method to data describing U.S. hospitals revealed nuanced differences in performance that are obscured in existing hospital rating systems.

## Introduction

Hospital performance can be characterized by an increasingly broad array of quality measures. The proliferation of these quality measures has given patients, policymakers and health care providers insight into many different domains of hospital quality, including patient experience, safety, care processes, and outcomes, such as mortality and readmission rates. However, the breadth of available quality measures makes it challenging to profile an individual hospital’s performance and to understand how it compares with others. There are several well-known ratings systems in the United States (U.S.) (i.e., U.S. News and World Report, Consumer Reports, Health Grades, and The Leapfrog Group) that produce a single aggregate performance rating, often reported as a number or letter grade, for each hospital.[1–4] However, these systems may obscure important differences in performance. For example, a hospital that offers a highly-rated patient experience but has poor outcomes and a hospital in which patients rate the experience as poor but have good outcomes may both be classified as average performers even though their performance in these key domains of hospital quality are quite different.

Characterizing the performance of an individual hospital across the full spectrum of quality measures generates a performance profile that cannot be easily communicated by ordinary hospital ranking systems or summary measures. While these ranking systems do provide some insight into aggregate hospital performance, new approaches for characterizing precise hospital performance in a way that preserves the nuance contained individual quality measures have not been described. Improved insight into hospital performance has the potential to better inform patient choice, guide efforts to improve quality, promote collaborations, and stimulate research into the key determinants of hospital quality. Just as precision medicine requires better phenotyping of individuals, quality improvement efforts might be enhanced by customized approaches that leverage a more precise characterization of the hospital performance profile.

Accordingly, our objective was to characterize hospital performance in the U.S. across a broad set of publicly-reported quality measures by describing certain commonly-occurring hospital performance profiles and to quantify differences between hospitals in a manner that is informative and accessible. To do this, we used a novel semi-supervised machine learning technique to produce a diffusion map that enables the visualization of similarities and differences between hospitals’ performance. This graph analytic approach uses distance to indicate differences in hospital performance: hospitals sharing a similar performance profiles, taking into account their performance on all the measures, are localized in space, while those with distinct performance profiles are separated by longer distances.

## Materials and methods

### Sample construction

Most hospitals in the U.S. are required to report their performance on various quality measures to the country’s primary public payor, the Centers for Medicare & Medicaid Services (CMS). These data are publicly reported via the Hospital Compare website, [5] which includes more than 100 measures spanning several domains of hospital quality that describe structural characteristics of hospitals (e.g., presence of cardiac surgery registry), care processes (e.g., proportion of patients with ST-segment elevation myocardial infarction who received primary percutaneous coronary intervention within 90 minutes of hospital arrival), patient experience, volume of certain inpatient and ambulatory procedures, value (e.g., proportion of patients who received cardiac stress testing before low-risk outpatient surgery), safety (e.g., rate of catheter-associated urinary tract infections), quality of surgical care (e.g., complication rates after hip and knee surgery), and risk-standardized readmission rates and risk-standardized mortality rates. The June 2014 data release was used in this analysis. To reflect our focus on hospital performance, certain measures that did not directly characterize quality were excluded (Table A in S1 File). Since our analytic methods are best suited to complete or near-complete data, we restricted our analysis to those hospitals that reported at least 90% of these quality measures and then excluding any measure that was reported by fewer than 90% of these hospitals. The resulting sample consisted of 1,614 (33.2%) of the 4,861 hospitals that consistently reported data to CMS across 84 quality measures.

### Hospital characteristics

We collected data that describe hospital characteristics, the demographics of the patients that they serve, and their performance on 4 popular hospital rating systems. Specifically, we used the 2013 American Hospital Association Annual Survey to identify hospital region (Northeast, South, Midwest, West), location (rural, urban), teaching status, and number of beds.[6] To characterize the demographics of the patient population that each hospital serves, we measured the proportion of the local population that was minority (i.e., non-white) and the median household income by calculating the weighted average of these 2 characteristics for all Zip Code Tabulation Areas that comprise a hospital’s Hospital Service Area, or local market using data from the U.S. Census.[7, 8] Lastly, we identified “top ranked” hospitals in 4 well-known hospital rating systems: U.S. News and World Report Best Hospital Rankings, HealthGrades Top Hospitals, Consumer Reports Hospital Ratings, and the Leapfrog Hospital Survey.[1–4] To do this, we used an established definition of “top ranked” hospitals, which identified up to 6% of hospitals included in our samples as top performers (Table C in S1 File).[9]

### Data organization and average performance profile

The characterization of hospital performance across a wide range of quality measures is effectively an organization problem. We developed a novel analytic approach that builds upon established semi-supervised machine learning methods to turn this originally unsupervised organization problem into a problem of building the optimal features for regressing hospital quality and depicting the result in an accessible format. Since the development and derivation of our mathematical methods are comprehensively described elsewhere, [10] this manuscript provides a general overview of these methods and focuses on their application to data describing hospital performance.

First, we standardized the data describing hospitals’ performance on each included quality measure to a normal distribution with a mean of 0 and standard deviation of 1. Next, we set any data value that was 4 standard deviations above or below the mean to these maximum and minimum values to produce “performance profiles” that characterize the performance of individual hospitals across all measures and “measure profiles” that characterize the range of performance for all hospitals for an individual measure.

Next, we used a coupled diffusion process to simultaneously organize these performance profiles and measure profiles by iteratively identifying correlations between these profiles of data.[11,12] Our method creates an iterative co-clustering of the quality measures and hospitals: it begins by building a diffusion metric on the quality measures, using cosine affinity between the measures, and then uses this metric to construct a coupled diffusion metric on the hospitals using an approximate earth mover’s distance between the hospitals. The algorithm then iterates between organizing the quality metrics and organizing the hospitals, building successively more reliable diffusion metrics.

Then, we constructed a binary partition tree on the space of low-frequency eigenvectors of the hospital diffusion metric to identify groups of comparable hospitals. We computed an average score on each quality measure for each of these groups. This produced an illustrative performance profile that represents the average performance of hospitals in each group. These illustrative performance profiles would serve as reference points to facilitate the creation of our diffusion map. Since each level of the partition tree was refined dyadically into two smaller clusters, we pre-specified that our model should produce 32 illustrative performance profiles, reasoning that this number of performance profiles would reflect the full spectrum of hospital performance and avoid excessive model supervision.

### Reference points and expert input

In the quality measures reported by CMS, there are many more measures describing processes and patient experience than measures in other domains that may be more important to patients, such as safety, readmission and mortality. The application of many analytic techniques, including traditional clustering and unsupervised machine learning methods, would treat each quality measure equally, meaning that their output would likely overemphasize the domains of hospital performance with the greatest number of measures. To avoid this problem, existing hospital rating systems assign weights to individual measures and domains. In our description of our mathematical methods, [10] we demonstrate that in the absence of supervision, the classification of performance profiles is coarse, with poor differentiation between hospitals with distinct performance. Consequently, we used input from three authors with extensive experience in quality measurement and also trained in emergency medicine, pediatrics, internal medicine, and cardiology, to supervise our algorithm, thereby improving its ability to discriminate between hospital performance profiles. These experts were asked to rank the 32 illustrative performance profiles, which were produced by the partition trees analysis described earlier, on a scale from 1 to 10 according overall hospital performance, with 10 representing top performance, by applying several pre-specified principles (Figure A in S1 File). For example, experts were told to favor consistent performance over outliers and to adopt a relative hierarchy of domains that is consistent with the CMS value-based purchasing program weights, in which outcomes are weighted more heavily than patients’ experience, which is weighted more heavily than process measures. Experts made their rankings independently and were blinded to each other’s rankings. Once responses from all experts were received, their rankings were compared. When there was disagreement about the ranking of an illustrative performance profile, some or all of the experts were asked to re-rank certain profiles using the profiles of performance for which agreement had already been established as benchmarks. The resulting ranking of the 32 illustrative performance profiles was used to orient our visualization (Figure B in S1 File). Importantly, this expert input does not represent a “gold standard” of what constitutes top performance; rather, it simply serves to supervise our model to ensure that it produces a pragmatic output.

### Diffusion map

To visualize how the performance of individual hospitals related to one another, we used diffusion mapping to plot each hospital as a point in multi-dimensional space with the distance between each point (hospital) representing the similarity of the underlying performance profile; the methods used to produce these diffusion maps are described in detail elsewhere.[10] Hospitals that are close to each other share similar performance profiles, while those that are far apart have distinct performance profiles. The diffusion mapping process used to create the visualization is a multi-step process that we have described previously.[10]

First, we used an ensemble of artificial neural networks to generate a high-dimensional set of features for each hospital that can be used to estimate a quality score for each hospital according to its distance from the 32 reference points. An artificial neural network is a set of nonlinear projections that take high-dimensional data to a low-dimensional representation that is optimized for regressing some function of interest, in this case the roughly propagated quality score. This redefines the spatial relationships between each hospital and the reference points in a non-Euclidean way. Subsequently, we used the hidden layer features of the neural network to build a non-Euclidean distance metric between any two hospitals, and a heat kernel that defines the degree of similarity between any two points based on how close they are in this neural network representation.

Next, we used the output of this kernel to plot a “diffusion map,” which projects the high-dimensional data onto 3-dimensional space, and which serves as the basis of all of the visualizations presented in this study. Since the appearance of the resulting visualizations can vary according to the dimensions chosen for projection, all visualizations presented in this paper are taken from the same vantage point in the same dimensions for consistency.

### Commonly-occurring performance profiles and statistical analysis

To identify commonly-occurring profiles of hospital performance, we identified neighborhoods of hospitals that shared similar patterns of performance and characterized the performance of a typical hospital in such neighborhoods across the full range of quality measures. To do this, we applied the model of "heat diffusion,” which involves repeatedly modeling the application of multiple heat sources to different locations of the diffusion map and the resulting diffusion time as this heat spreads across the entire surface of the diffusion map. The configuration of heat sources that spreads most quickly (i.e., reaches every point in the smallest time) can then be used to define areas of closely related points with comparable local variation in their performance profiles. We pre-specified that we would define 16 neighborhoods, reasoning that approximately 100 hospitals would be classified into each neighborhood given our sample size. Correspondingly, we applied 16 heat sources to our diffusion map to identify “neighborhoods” of hospitals that performed similarly on the quality measures, labeling each with a letter (A through P) for ease of description. To characterize the performance profile that defined each neighborhood, we identified the central hospital (i.e., site of heat application) and its 10 nearest neighbors before calculating the average of their performance on each of the included quality measures.

### Statistical analysis

Descriptive statistics were used to compare the characteristics of hospitals included in our sample to the broader population of hospitals for quality measures were publicly reported by CMS. Similarly, we used descriptive statistics to characterize the structural features of all hospitals that comprise each neighborhood, as well as the hospital service area demographics and the presence of hospitals designated as high performers under existing rating systems. All statistical analysis was performed in SAS version 9.3; the maps were produced with MATLAB 2015b. This study used publicly available data and was therefore exempt from approval by the Yale University Institutional Review Board.

## Results

### Sample construction

Hospitals included in our sample tended to be larger: the proportion of hospitals with fewer than 100 beds was 3.6% compared with 50.4% for the broader population of U.S. hospitals for which quality measures were publicly reported (Table B in S1 File). The geographic distribution of hospitals included in our sample was comparable to that of the broader population; however, our sample contained few rural hospitals. Additionally, our sample had a higher proportion of teaching hospitals, and the median household income in each hospital’s local market, known as “hospital service areas,” was slightly higher for hospitals included in our sample than that among the overall population of hospitals in the U.S. The median proportion of the population in each hospital service area that was a racial minority was also significantly higher in our sample. The vast majority of hospitals that were top-ranked by U.S. News and World Report (16 of 17; 94.1%) and HealthGrades (93 of 100; 93.0%) were included in our sample. In contrast, only 31.0% (36 of 116) of hospitals that were top-ranked by Consumer Reports and 37.3% (31 of 83) of top-ranked hospitals by The Leapfrog Group were included (Table C in S1 File).

### Mapping hospital performance

**Fig 1:**
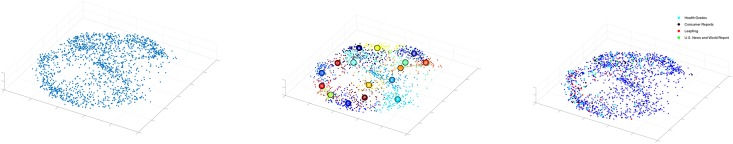
Diffusion maps showing all hospitals (A), hospitals labelled according to their performance profile (B) and top-rated hospitals (C).

The diffusion map localizes hospitals according to the pattern of their performance across all of the measures (Fig 1A and S1 Movie). There is a high density of hospitals in certain parts of the diffusion map, which indicates that there are groups of hospitals that share similar performance profiles, while there are few hospitals in other parts, suggesting that certain performance profiles are rare. We identified 16 distinct neighborhoods of hospitals (Fig 1B) that contained between 44 and 196 hospitals (median number of hospitals per neighborhood, 90), and describe the performance profile that characterizes the central hospitals in each neighborhood. Hospitals that were top-ranked by the 4 well-known hospital rating systems were generally distributed broadly across the map (Fig 1C).

### Commonly-occurring hospital performance profiles

**Fig 2:**
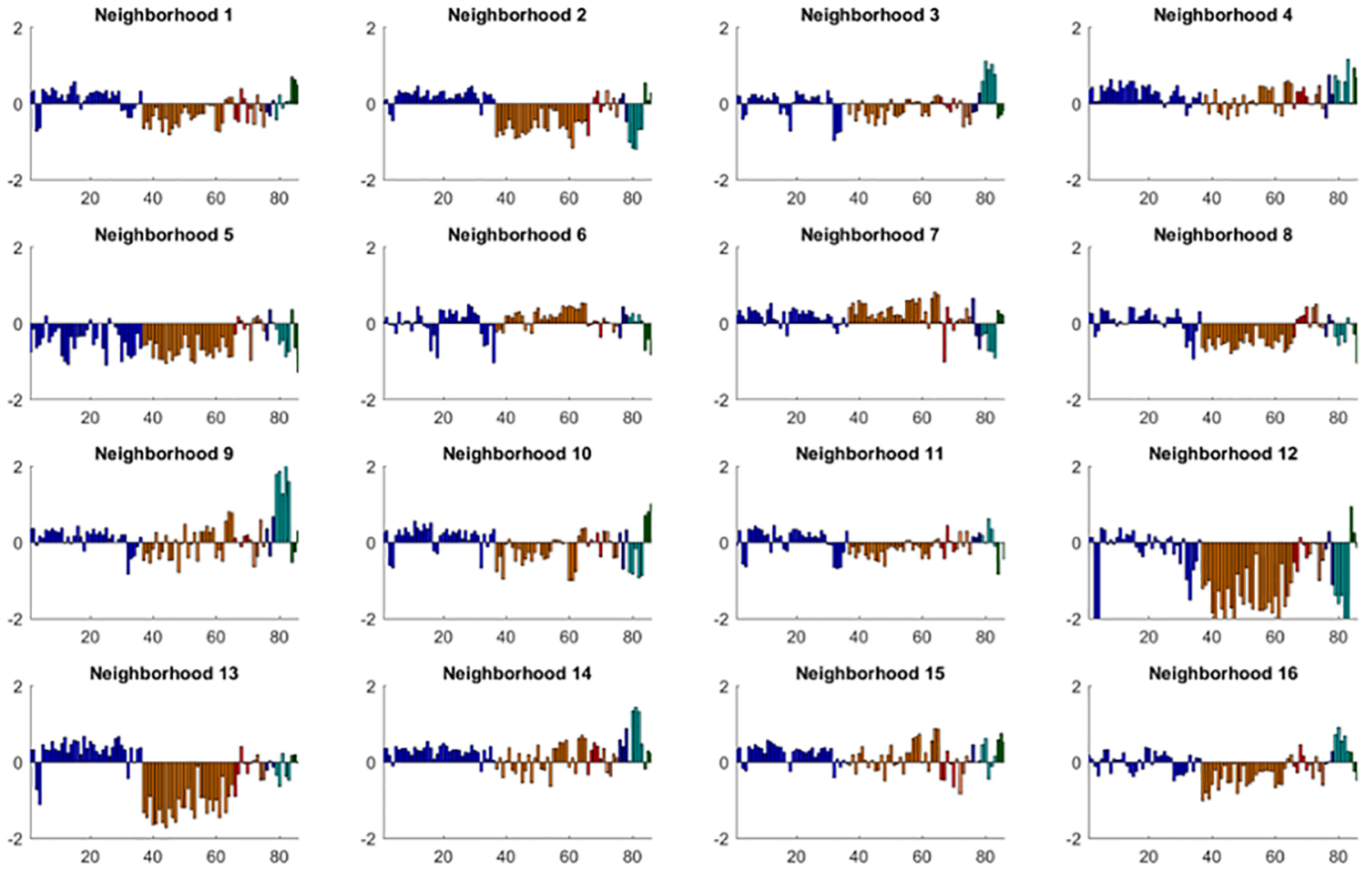
Performance profiles of the 16 hospital performance profiles. The vertical bars represent the average normalized score for the central hospital in each neighborhood and its 10 nearest neighbors on the 84 quality measures. The vertical scale is standard deviations from the mean. The bars are grouped and shaded according to the domain of the quality measure (blue: process, orange: experience, red: value, purple: safety, navy: surgery, turquoise: readmission, green: mortality).

The performance profiles that define each of the 16 neighborhoods were distinct (Table 1, Fig 2, and S2 Movie). The performance profile of neighborhood D was the most consistent: scores were positive (i.e., above the mean) in all 7 measurement domains and this neighborhood had the highest score on 11 process measures, the lowest composite rate of surgical complications, and generally good performance on both readmission and mortality measures, especially for pneumonia. The performance profile of neighborhood N was defined by excellence in orthopedic surgery: the rate of complication and readmission after hip and knee surgery was the lowest of all neighborhoods. Best-in-class experience distinguished the performance profile of neighborhood G, which had the highest score on just over half of the measures of patient experience. Hospitals in neighborhood E had a performance profile that was characterized by below-average scores in many domains, with the lowest score in 23 of 30 process measures, the highest rate of central line-associated blood stream infections, death among patients with treatable complications of surgery, and 30-day mortality after hospitalization for acute myocardial infarction and pneumonia. Similarly, neighborhood L had a performance profile that was characterized by a poor patient experience, high rates of methicillin-resistant *Staphylococcus Aureus* bloodstream infections, complications after hip and knee surgery, and readmissions, although hospitals in this neighborhood had the lowest rate of 30-day mortality after heart failure admissions. The performance profile characterizing neighborhood I had the lowest rates of readmission for acute myocardial infarction, heart failure, and pneumonia; however, performance on mortality measures was worse than that of other neighborhoods. Comparisons of commonly-occurring profiles demonstrate that the relationships between individual measures and domains is complicated. For example, the performance profile of hospitals in neighborhoods B and G share common features such as low mortality rates and high rates of readmission; however, their performance on patient experience measures was sharply divergent.

**Table 1:** Characteristics of hospitals, their hospital service area demographics, and presence of high-performing hospitals in each neighborhood.

Neighborhood	Process	Experience	Value	Safety	Surgery	Readmission	Mortality
A (N = 196)	Good	Poor	Mixed	Mixed	Poor	Mixed	Good
B (N = 138)	Good (highest score on 3 measures)	Poor	Mixed	Mixed	Mixed	Poor, with particularly high readmission for AMI, HF, and Hip & knee surgery	Good
C (N = 135)	Mixed (highest score on 1 measure)	Mixed	Average, with lowest rate of overall spending, but highest use of with/without contrast CT	Poor, with highest rate of surgical site infection from colon surgery	Mixed	Good, with particularly low readmission for HF, and Hospital-wide	Poor
D (N = 117)	Good (highest score on 11 measures, but lowest on 1)	Mixed	Good with lowest use of chest CT with and without contrast	Average	Mixed, with lowest composite rate of surgical complications	Good, with particularly low readmission for Pneumonia	Good, with lowest mortality for Pneumonia
E (N = 155)	Poor (lowest score on 23 measures)	Poor	Average	Mixed, with highest rate of CLABSI	Mixed, with highest rate of death among patients with treatable complications of surgery	Poor	Mixed, with highest mortality for Pneumonia, and AMI
F (N = 140)	Mixed (highest score on 2 measures, lowest on 4)	Good (highest score on 7 measures)	Average	Average	Mixed	Good	Poor
G (N = 113)	Good (highest score on 4 measures)	Good (highest score on 16 measures)	Mixed, with highest mammography recall rate	Good, with lowest rate of C. Difficile infections	Mixed with lowest rate of death among patients with treatable complications of surgery	Poor	Good
H (N = 87)	Mixed (highest score on 1 measure, lowest on 1)	Poor	Mixed, with lowest rate of stress tests before low-risk ambulatory surgery	Good, with lowest rates of: CAUTI, and surgical site infection from colon surgery	Mixed	Poor	Poor, with particularly high mortality for AMI
I (N = 65)	Mixed (highest score on 2 measures)	Mixed (highest score on 2 measures)	Average, with highest use of abdominal CT with and without contrast	Mixed, with lowest rate of surgical site infection after hysterectomy	Mixed	Good, with lowest readmission rates for AMI, HF, Pneumonia, and Hospital-wide	Poor
J (N = 93)	Mixed	Poor (lowest score on 1 measure)	Mixed	Mixed	Mixed, with highest composite rate of surgical complications	Poor	Good, with lowest mortality for AMI
K (N = 65)	Mixed (highest on 1 measure)	Poor	Mixed	Mixed	Good	Mixed	Poor, with highest mortality for HF
L (N = 44)	Mixed (lowest score on 5 measures)	Poor (lowest score on 23 measures)	Poor	Poor, with highest rate of MRSA bloodstream infections	Mixed, with highest rate of complications after hip & knee surgery	Poor, with highest readmission rates for AMI, HF, Pneumonia, Hip & knee, and Hospital-wide	Good, with lowest mortality for HF
M (N = 71)	Good (highest score on 14 measures)	Poor (lowest score on 5 measures)	Poor, with highest overall spending	Mixed	Average	Poor	Average
N (N = 62)	Good (highest score on 5 measures)	Mixed (lowest score on 1 measure)	Good, with lowest mammography recall rate and use of abdominal CT with and without contrast	Mixed, with lowest rate of CLABSI	Good, with lowest rate of complications after hip & knee surgery	Good, with lowest readmission after hip & knee surgery, but hospital-wide and HF readmission also low	Average
O (N = 58)	Good (highest on 4 measures)	Good (highest on 4 measures)	Poor, with highest rate of stress tests before low-risk ambulatory surgery	Poor, with highest rate of CAUTI	Good	Mixed	Good
P (N = 75)	Mixed (highest on 1 measure, lowest on 2 measures)	Poor (n.b., particularly poor performance on cleanliness)	Mixed	Mixed, with highest rate of C. Difficile infections	Average	Good	Mixed

AMI, acute myocardial infarction; CAUTI, catheter-associated urinary tract infection; CLABSI, central line-associated blood stream infection; HF, heart failure; MRSA, Methicillin-resistant *Staphylococcus aureus*

Definitions: Performance within a domain was classified as good if most measures were above average (i.e., 0), as poor if most measures were below average, as mixed if multiple measures were both greater than and less than average, and as average if most measures were near the average. The neighborhoods with the best and worst performance for each measure are noted. Neighborhoods with particularly good or bad performance, defined as a value 1 standard deviation above or below the average, on any measure were also noted.

### Performance profiles and hospital characteristics

While neighborhoods were defined by commonly-occurring performance profiles, there were some similarities and some differences in the characteristics of the hospitals comprising each neighborhood (Table 2). For example, the proportion of teaching hospitals was particularly high in neighborhoods A, I, and J, and the performance profile of these neighborhoods was mixed, with good performance on either the mortality or readmission measures but not both. Smaller hospitals tended to cluster in neighborhood E, for which the performance profile indicated generally poor performance across many measurement domains, although smaller hospitals were also common in neighborhoods F and G where performance was better. Hospitals in neighborhoods B, H, and M tended to serve a high proportion of non-white minority patients: the median proportion of non-white patients residing in the corresponding hospital service area was 27.6% (IQR: 12.9%-41.4%), 28.3% (IQR: 19.0%-43.0%) and 32.9% (21.1%-52.6%) respectively, and the performance profiles of these neighborhoods indicated that readmission rates were higher in these neighborhoods. The median household income across the local hospital service area was highest in neighborhood D and the associated performance profile indicated strong performance across all domains.

**Table 2:** Summary characteristics of hospitals comprising each neighborhood, demographics of their Hospital Service Areas, and their U.S. News and World Report, Leapfrog, Consumer Reports, and Health Grades ratings.

Neighborhood	A	B	C	D	E	F	G	H	I	J	K	L	M	N	O	P	P-value
Number of hospitals	196	138	134	117	154	139	113	86	65	93	65	44	71	62	57	75	
**Region**																	<0.001
Northeast	59	44	15	14	22	14	18	11	5	28	9	18	24	6	15	11	
	30.1%	31.9%	11.2%	12.0%	14.3%	10.1%	15.9%	12.8%	7.7%	30.1%	13.9%	40.9%	33.8%	9.7%	26.3%	14.7%	
South	41	40	32	32	30	31	46	14	18	39	17	8	4	22	15	17	
	20.9%	29.0%	23.9%	27.4%	19.5%	22.3%	40.7%	16.3%	27.7%	41.9%	26.2%	18.2%	5.6%	35.5%	26.3%	22.7%	
Midwest	55	43	43	33	72	87	44	44	16	17	31	11	26	21	19	32	
	28.1%	31.2%	32.1%	28.2%	46.8%	62.6%	38.9%	51.2%	24.6%	18.3%	47.7%	25.0%	36.6%	33.9%	33.3%	42.7%	
West	41	11	44	38	30	7	5	17	26	9	8	7	17	13	8	15	
	20.9%	8.0%	32.8%	32.5%	19.5%	5.0%	4.5%	19.8%	40.0%	9.7%	12.3%	15.9%	23.9%	21.0%	14.0%	20.0%	
**Location**																	
Urban	196	137	133	115	154	137	112	86	64	93	64	44	71	62	57	75	
	100.0%	99.3%	99.3%	98.3%	100.0%	98.6%	99.1%	100.0%	98.5%	100.0%	98.5%	100.0%	100.0%	100.0%	100.0%	100.0%	
Rural	0	1	1	2	0	2	1	0	1	0	1	0	0	0	0	0	
	0.0%	0.7%	0.7%	1.7%	0.0%	1.4%	0.9%	0.0%	1.5%	0.0%	1.5%	0.0%	0.0%	0.0%	0.0%	0.0%	
**Bed Size**																	<0.001
<100	5	1	4	7	10	11	5	3	1	0	4	0	2	1	0	4	
	2.6%	0.7%	3.0%	6.0%	6.5%	7.9%	4.4%	3.5%	1.5%	0.0%	6.2%	0.0%	2.8%	1.6%	0.0%	5.3%	
100–199	37	31	37	32	59	40	41	21	12	20	14	12	16	11	15	26	
	18.9%	22.5%	27.6%	27.4%	38.3%	28.8%	36.3%	24.4%	18.5%	21.5%	21.5%	27.3%	22.5%	17.7%	26.3%	34.7%	
200–299	50	37	41	22	44	37	26	24	15	25	17	15	15	16	18	19	
	25.5%	26.8%	30.6%	18.8%	28.6%	26.6%	23.0%	27.9%	23.1%	26.9%	26.2%	34.1%	21.1%	25.8%	31.6%	25.3%	
300–399	42	26	21	25	13	26	14	15	13	14	12	7	18	17	10	10	
	21.4%	18.8%	15.7%	21.4%	8.4%	18.7%	12.4%	17.4%	20.0%	15.1%	18.5%	15.9%	25.4%	27.4%	17.5%	13.3%	
≥400	62	43	31	31	28	25	27	23	24	34	18	10	20	17	14	16	
	31.6%	31.2%	23.1%	26.5%	18.2%	18.0%	23.9%	26.7%	36.9%	36.6%	27.7%	22.7%	28.2%	27.4%	24.6%	21.3%	
**Teaching**																	<0.001
Yes	117	64	63	64	55	55	53	37	39	59	32	24	33	32	31	33	
	59.7%	46.4%	47.0%	54.7%	35.7%	39.6%	47.3%	43.0%	60.0%	63.4%	49.2%	54.6%	46.5%	51.6%	54.4%	44.0%	
No	79	74	71	53	99	84	59	49	26	34	33	20	38	30	26	42	
	40.3%	53.6%	53.0%	45.3%	64.3%	60.4%	52.7%	57.0%	40.0%	36.6%	50.8%	45.5%	53.5%	48.4%	45.6%	56.0%	
**Average proportion of Hospital Service Area population that is non-white**																	<0.001
Median	21.9%	27.6%	14.6%	20.1%	23.3%	17.9%	19.1%	28.3%	15.0%	23.6%	22.6%	26.6%	32.9%	18.0%	20.0%	20.4%	
Q1	12.5%	12.9%	10.5%	11.6%	11.9%	9.5%	10.9%	19.0%	9.3%	12.6%	12.5%	16.3%	21.1%	11.7%	11.0%	8.7%	
Q3	34.3%	41.4%	23.6%	30.4%	36.1%	29.6%	31.5%	43.0%	24.8%	33.5%	33.5%	48.6%	52.6%	28.6%	27.1%	29.7%	
**Average household income in Hospital Service Area**																	<0.001
Median	$ 58,709	$ 56,083	$ 50,443	$ 61,928	$ 48,233	$ 48,954	$ 51,049	$ 52,004	$ 52,729	$ 57,673	$ 53,666	$ 54,929	$ 56,083	$ 58,526	$ 59,200	$ 50,624	
Q1	$ 50,338	$ 46,298	$ 44,571	$ 56,250	$ 41,723	$ 40,760	$ 43,803	$ 44,927	$ 45,619	$ 48,632	$ 45,839	$ 48,617	$ 47,039	$ 48,632	$ 50,082	$ 45,551	
Q3	$ 76,021	$ 65,378	$ 58,788	$ 68,198	$ 56,558	$ 57,483	$ 61,086	$ 61,148	$ 58,956	$ 71,865	$ 59,654	$ 66,531	$ 70,857	$ 67,873	$ 72,125	$ 58,788	
**High performers in existing hospital rating systems**																	
U.S. News and World Report																	
Number	6	1	0	2	2	0	3	1	0	1	0	0	0	0	0	0	
%	37.5%	6.3%	0.0%	12.5%	12.5%	0.0%	18.8%	6.3%	0.0%	6.3%	0.0%	0.0%	0.0%	0.0%	0.0%	0.0%	
Health Grades																	
Number	14	8	9	18	3	0	4	3	7	8	2	0	0	9	5	3	
%	15.1%	8.6%	9.7%	19.4%	3.2%	0.0%	4.3%	3.2%	7.5%	8.6%	2.2%	0.0%	0.0%	9.7%	5.4%	3.2%	
Consumer Reports																	
Number	4	0	3	12	2	1	0	0	5	1	0	0	0	5	3	0	
%	11.1%	0.0%	8.3%	33.3%	5.6%	2.8%	0.0%	0.0%	13.9%	2.8%	0.0%	0.0%	0.0%	13.9%	8.3%	0.0%	
Leapfrog																	
Number	5	3	3	4	1	1	2	1	2	2	0	0	1	4	1	1	
%	16.1%	9.7%	9.7%	12.9%	3.2%	3.2%	6.5%	3.2%	6.5%	6.5%	0.0%	0.0%	3.2%	12.9%	3.2%	3.2%	

### Performance on existing rating systems by neighborhood

Top-ranked hospitals were found in 15 of the 16 neighborhoods, with no neighborhood containing more than 40% of the top performers under a single hospital rating system. Despite the diffuse appearance, there was some clustering of top-ranked hospitals in certain neighborhoods. For example, top performing hospitals according to U.S. News and World Report and Leapfrog were particularly concentrated in neighborhood A. Top performers in the Health Grades and Consumer Reports rankings were particularly concentrated in neighborhood D that had a consistent performance profile with above average performance in all domains. The presence of other hospitals adjacent to top-ranked hospitals on the diffusion map indicate that there may be several hospitals with performance profiles comparable to those of top-ranked hospitals that were not acknowledged by existing rating schemes.

## Discussion

We produced a diffusion map of hospitals in the U.S. that describes hospital performance profiles, thereby introducing an approach to precisely characterize hospital performance across a wide range of publicly-reported quality measures. This approach retains the nuances of similarities and differences in hospital performance across the range of quality measures. To do this, we developed a graph analytic, semi-supervised machine learning technique, guided by input from experts in quality measurement, to organize hospitals according to the totality of their performance on the full range of quality measures released by CMS. The resulting visualization, which provides a graphical and quantitative characterization of hospital performance, could form the basis of a new tool for communicating the differences between hospitals that are often lost in subjective reviews or existing hospital rating systems and may even provide a better basis for incentive programs and improvement initiatives.

Traditional classification approaches, which are used by some existing hospital rating systems, are reductionist. These approaches may group hospitals that have comparable “overall” performance yet perform quite differently on certain quality measures together. When top-ranked hospitals from 4 contemporary hospital rating systems were highlighted on our diffusion map, we identified many other hospitals that shared similar performance profiles as the top-ranked hospitals yet were not classified as top-ranked hospitals themselves. In addition, top-ranked hospitals tended to be diffusely distributed across our map, suggesting that there are important differences in overall performance profile that are not reflected by the existing hospital rating schemes, even though the same publicly available quality measures used in this study are incorporated into these ratings.

The performance profile of hospitals contained in each of the 16 neighborhoods differed in subtle but important ways. These differences may have important implications for patients and other stakeholders. For example, we identified 2 neighborhoods (B and G) that share several similar features including high rates of adherence to process measures, low mortality rates, and high rates of readmission. However, our approach found that the patient experience differs markedly between hospitals in these 2 apparently similar neighborhoods. Although not all patients can choose the hospital where they will receive acute care, such nuanced insights into differences between hospitals can enable those patients who do have a choice to select a hospital that fits their personal priorities and needs. Hospitals with certain characteristics, for example a small number of beds, tended to cluster in specific neighborhoods with distinct performance profiles.

Our study has several limitations. Since our analytic method is best suited to complete data sets, we focused on a sample of hospitals where reporting of the included quality measures was nearly universal. This approach to sample construction has 2 implications. First, it means that the sample of hospitals used to create our map are not necessarily representative of the broader population of hospitals in the U.S. because the hospitals in our sample tended to be larger and more frequently in urban areas. Second, we excluded some quality measures that were seldom reported, such as certain process measures. While this analysis focused on the measures reported through the Hospital Compare program, other data characterizing hospital quality, or more broadly the hospital environment, could readily be included. Finally, there are many ways to organize hospital performance data and we are not presenting the technique as a gold standard, but rather an effective way to summarize multiple measures across disparate domains.

In conclusion, we applied a graph analytic, machine learning technique to a dataset describing various established measures of hospital quality to produce a map of hospital quality reflecting each hospital’s performance across a wide range of quality measures. We found clusters of hospitals with distinct performance profiles and structural characteristics. An improved ability to characterize hospital performance could better inform patient choice, enhance understanding of what leads hospitals to be like others in their overall performance, and promote precision quality improvement approaches that are specific to a hospital’s particular profile.

## Supporting information

S1 File Contains supplementary Figures A and B, Tables A, B and C, and Movies A and B. (DOCX)

S1 Movie Overall diffusion map. (MP4)

S2 Movie Diffusion map in which each hospital is shaded according to its assigned neighborhood and the central hospital in each neighborhood is circled. (MP4)
