# Supplementary material for: Describing the performance of U.S. hospitals by applying big data analytics
Source: PLoS One. 2017 Jun 29;12(6):e0179603. doi: 10.1371/journal.pone.0179603 (PMC5491053; doi:10.1371/journal.pone.0179603)
Supplement: S1 File — (DOCX) [file pone.0179603.s001.docx]

S1 File: Supplementary Materials

Precision hospital performance: describing phenotypes by applying big data analytics

Nicholas S. Downing, Alexander Cloninger, Arjun K. Venkatesh, Angela Hsieh,

Elizabeth E. Drye, Ronald R. Coifman, Harlan M. Krumholz^*^

*Corresponding author

E-mail: harlan.krumholz@yale.edu (HMK)

**Fig A. Instructions provided to experts in quality measurement.**

| **Objective: To characterize similarities and differences in the profiles of performance of 32 performance profiles that synthesize the full spectrum of hospital performance on the Hospital Compare measures**  **Approach: Please follow this two-step process to define the anchors that supervise our model (see key principles for each step below)**   1. Review the profile of performance for each illustrative hospital and classify performance in each domain of quality 2. Assign an integer from 1 (lowest) to 10 (highest) that summarizes the overall performance of each illustrative hospitals across domains     **Step 1: Principles to inform the classification of performance by domain**   - Five categories of performance:   - ↑↑: Best performance in the domain across all illustrative hospitals (most measures in the domain, on average, are approximately 1 S.D. greater than the mean)   - ↑: Better performance in the domain (most measures in the domain, on average, fall between the mean and mean + 1 S.D.)   - Par: Average performance in the domain (most measures close to the mean, or similar proportions of measures above/below the mean)   - ↓: Worse performance in the domain (most measures in the domain, on average, fall between the mean and mean - 1 S.D.)   - ↓↓: Worst performance in the domain across all illustrative hospitals (most measures in the domain, on average, are approximately 1 S.D. less than the mean) - Classification should reflect the aggregate performance across the entire domain, favoring consistent performance over outliers - It is acceptable (and expected) that several performance profiles will have the same (or very similar) classifications across domains, and these hospitals can be grouped in the subsequent ranking step     **Step 2: Principles to inform the assignment of an integer summarizing overall performance**   - Relative hierarchy of domains is consistent with CMS’ FY17 Value-Based Purchasing weightings   - Mortality   - Experience//Readmission   - Surgical//Safety (HAI)//Value   - Process - It is acceptable for multiple illustrative hospitals to receive the same ranking, even if profiles of performance differ |
| --- |

Fig B. Materials provided to experts in quality measurement, including all 32 performance profiles generated from the coupled diffusion process and partition trees.


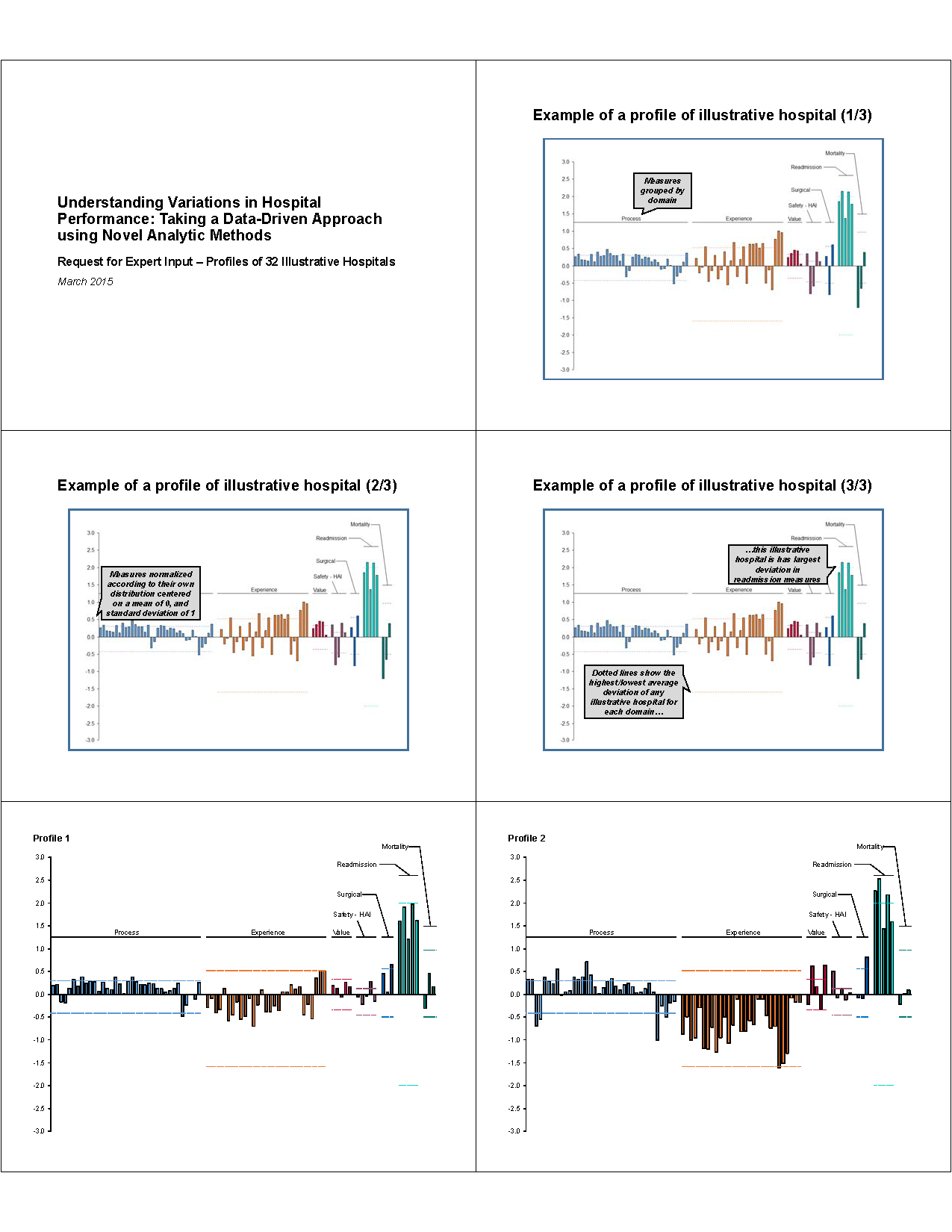


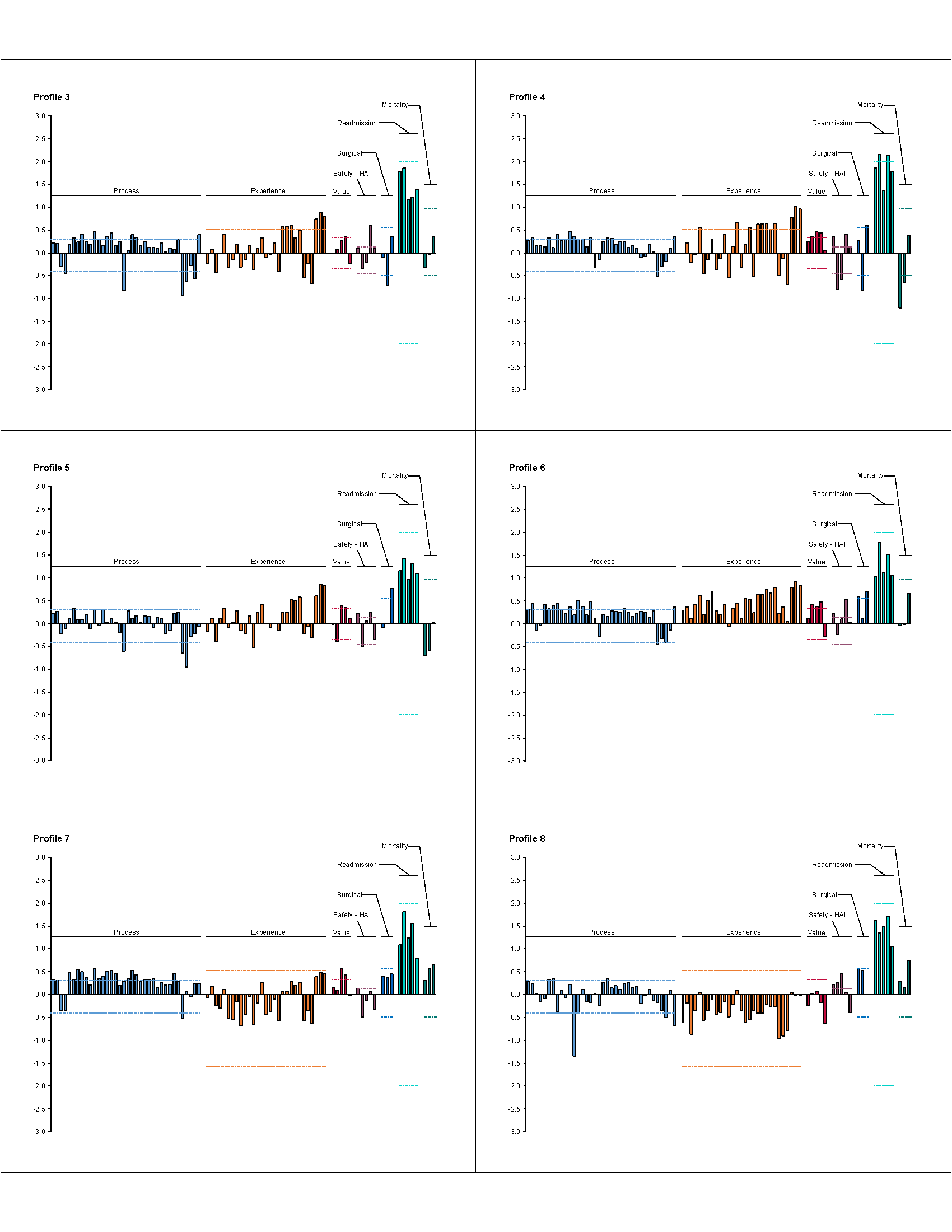


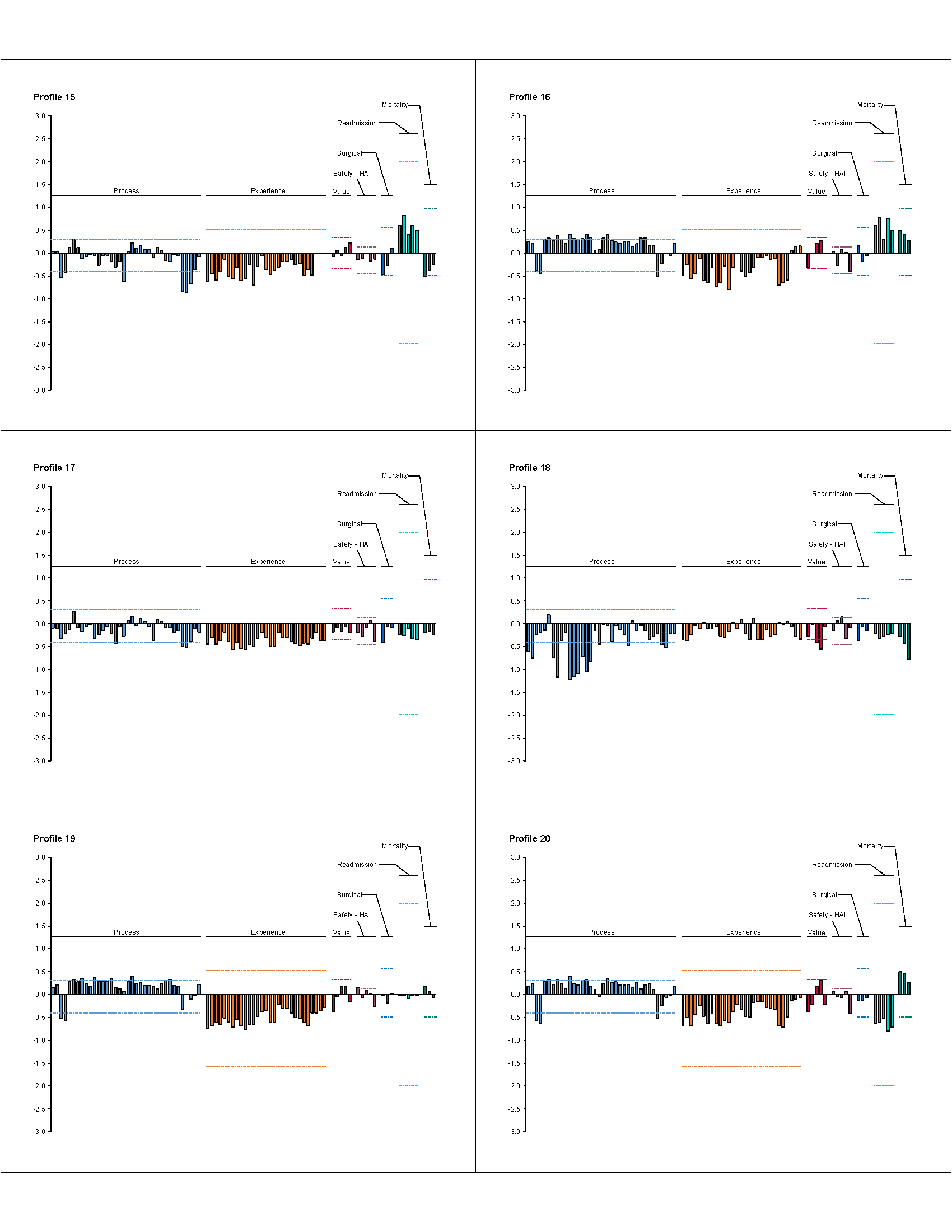


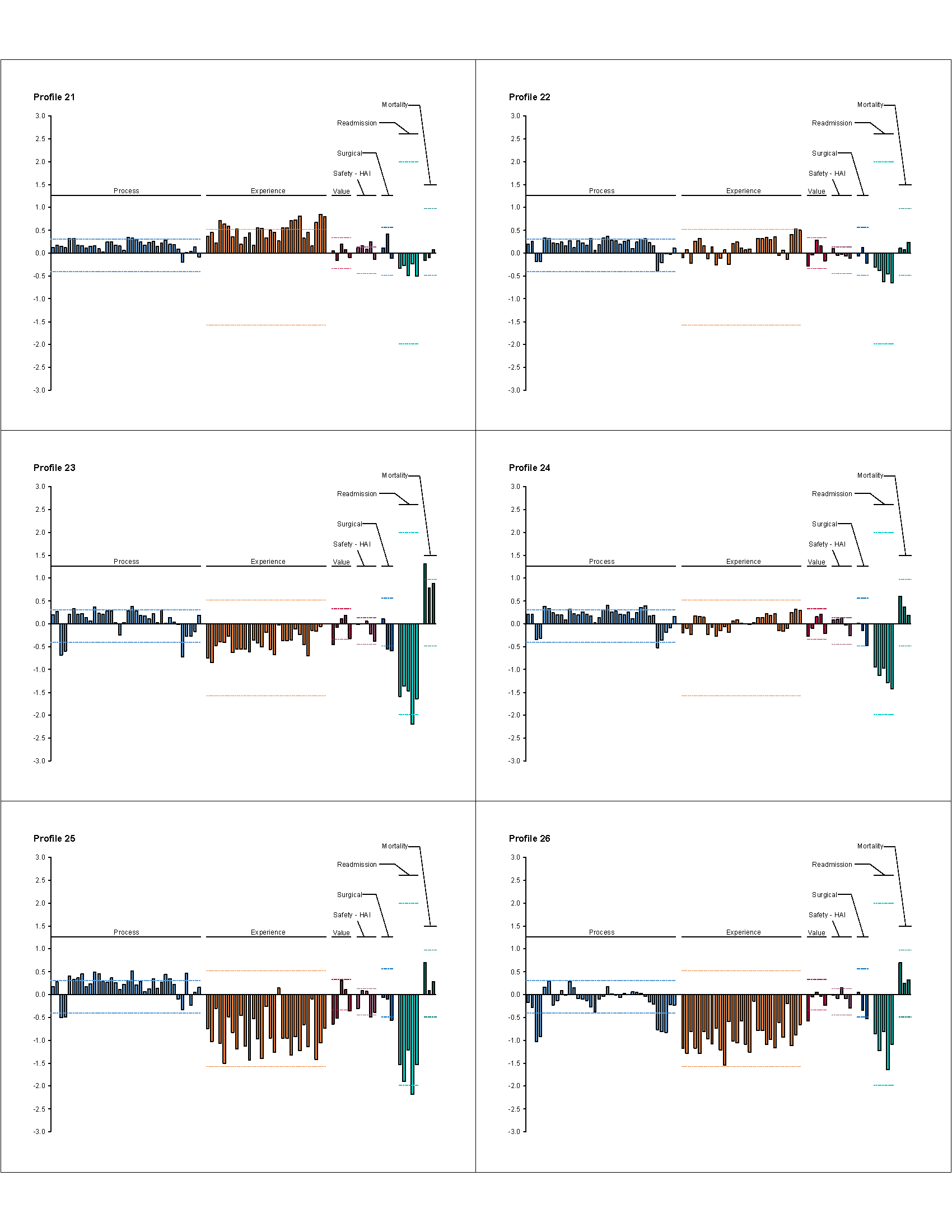


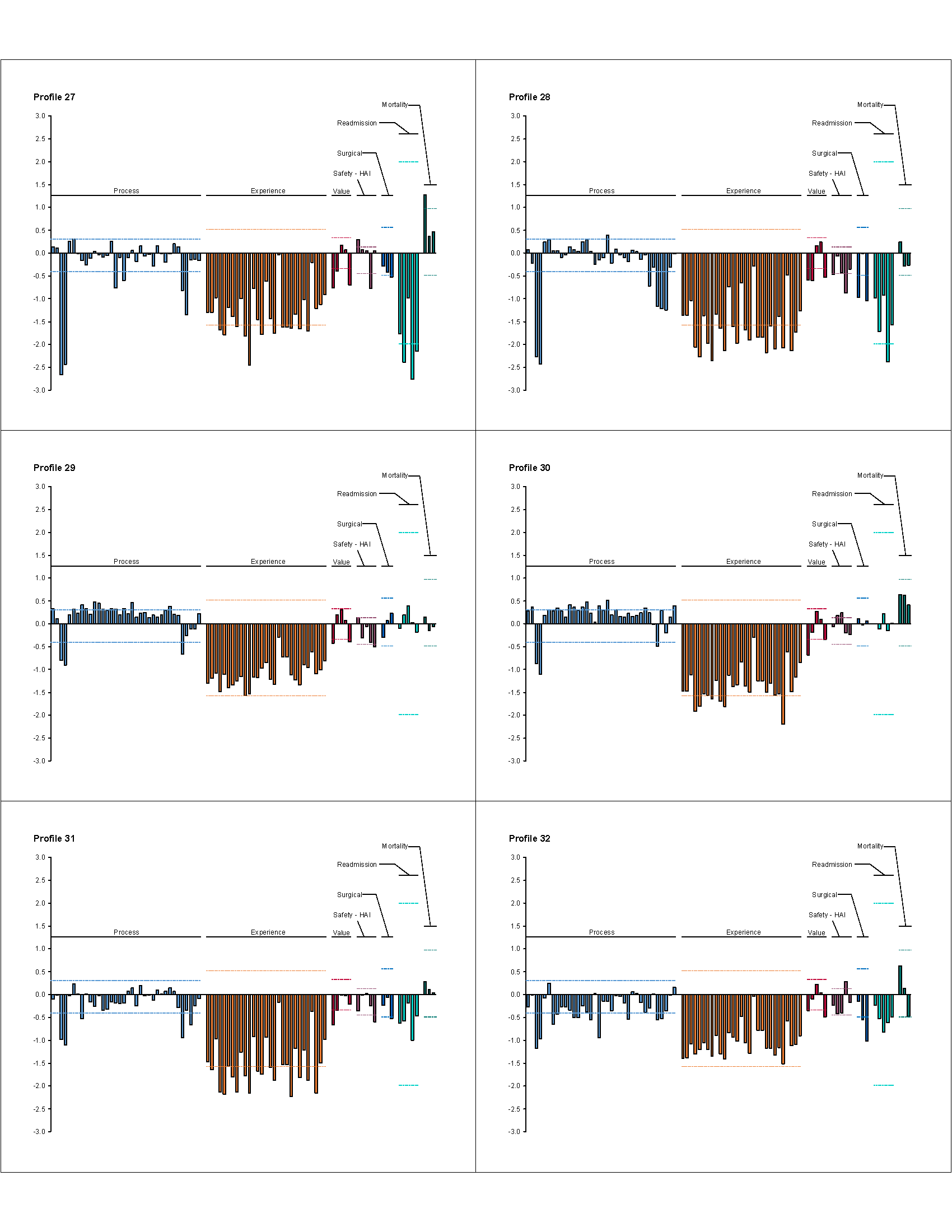


Table A. Excluded measures.

| *Structural measures* |
| --- |
| - OP_12: Ability of patients to check labs online |
| - OP_17: Ability of providers to track patients’ lab results, tests and referrals electronically between visits |
| - OP_25: Ability of providers to receive lab results electronically |
| - SM_PART_CARD: Presence of a cardiac surgery registry |
| - SM_PART_GEN_SURG: Presence of a general surgery registry |
| - SM_PART_STROKE: Presence of a stroke registry |
| - ACS_REGISTRY: Acute coronary syndrome registry |
| *Volume of selected outpatient procedures (OP_26_xx measures)* |
| - Gastrointestinal |
| - Eye |
| - Nervous system (i.e., CNS injections) |
| - Musculoskeletal |
| - Skin |
| - Genitourinary |
| - Cardiovascular |
| - Respiratory |
| *Reported at fewer than 5% of all Medicare hospitals* |
| - AMI_7a: Heart attack patients who got drugs to break up blood clots within 30 minutes of arrival |
| - OP_1: Median time to fibrinolysis |
| - OP_2: Outpatients with chest pain or possible heart attack who got fibrinolytic therapy within 30 minutes of arrival |
| - CAC_1: Children who received reliever medication while hospitalized for asthma |
| - CAC_2: Children who received systemic corticosteroid medication (oral and IV medication that reduces inflammation and controls symptoms) while hospitalized for asthma |
| - CAC_3: Children and their caregivers who received a home management plan of care document while hospitalized for asthma |

**Table B. Characteristics of hospitals, the demographics of their hospital services areas and high performers in existing rating systems.**

|  | **U.S. Hospitals**  **(N=4665)** | **Sample**  **(N=1609)*** |
| --- | --- | --- |
| **Hospital characteristics – no (%)** |  |  |
| Region |  |  |
| Northeast | 577 (12.4%) | 313 (19.5%) |
| South | 1378 (29.5%) | 406 (25.2%) |
| Midwest | 1745 (37.4%) | 594 (36.9%) |
| West | 914 (19.6%) | 296 (18.4%) |
| Other^†^ | 51 (1.1%) | 0 (0.0%) |
| Location |  |  |
| Urban | 3536 (75.8%) | 1600 (99.4%) |
| Rural | 1129 (24.2%) | 9 (0.6%) |
| Critical Access Hospital |  |  |
| Yes | 1251 (26.8%) | 0 (0.0%) |
| No | 3414 (73.2%) | 1609 (100.0%) |
| Size |  |  |
| <100 beds | 2351 (50.4 %) | 58 (3.6%) |
| 100-200 beds | 974 (20.9 %) | 424 (26.4%) |
| 200-300 beds | 552 (11.8%) | 421 (26.2%) |
| 300-400 beds | 332 (7.1%) | 283 (17.6%) |
| ≥400 beds | 456 (9.8%) | 423 (26.3%) |
| Teaching hospital |  |  |
| Yes | 1231 (26.4%) | 792 (49.2%) |
| No | 3434 (73.6%) | 817 (50.8%) |

| **Hospital service areas characteristics** | | |
| --- | --- | --- |
| Proportion of population that is racial minority – median (IQR) | 15.8% (6.8%-30.2%) | 21.1% (11.5%-33.5%) |
| Average household income – median (IQR) | $48,954 ($41,724 -$57,926) | $54,219 ($46,012-$64,233) |
| **High performers in existing hospital rating systems – no (%)** | | |
| U.S. News and World Report Honor Roll | 17 (0.4%) | 16 (1.0%) |
| HealthGrades Top 100 | 100 (2.1%) | 93 (5.8%) |
| Consumer Reports | 116 (2.5%) | 36 (2.2%) |
| Leapfrog “A” Grade | 83 (1.8%) | 31 (1.9%) |

*5 hospitals included in our sample were not listed in the 2013 American Hospital Association Annual Survey and could not be included in this table.

^†^ Puerto Rico, Guam, Virgin Islands, and American Samoa

**Table C. Definition of top ranked hospitals under four existing rating systems.**

| **Rating system** | **Definition of “high performer”** |
| --- | --- |
| Leapfrog | Recipient of Leapfrog Top Hospital award in 2014 (http://www.leapfroggroup.org/TopHospitals) |
| U.S. News and World Report | Listed on the U.S. News Honor Roll 2014-2015 (http://health.usnews.com/health-news/best-hospitals/articles/2014/07/15/best-hospitals-2014-15-overview-and-honor-roll) |
| Consumer Reports | Hospitals achieving a safety score of > 65, which corresponds to 2 standard deviations above the mean (http://www.consumerreports.org/health/doctors-hospitals/hospital-ratings.htm) |
| HealthGrades | America’s 100 Best Hospitals in 2014 (http://www.healthgrades.com/quality/2014-healthgrades-americas-best-hospitals-report) |

Adapted from definition presented by Austin MJ et al. *Health Affairs* 2015;34(3):423-430.

**Captions for 2 Movies:**

Two movies of the diffusion map have been produced. In these, each point represents an individual hospital. The distance between points reflects the similarity of their underlying performance profiles.

**Movie A.** Overall diffusion map.

**Movie B.** Diffusion map in which each hospital is shaded according to its assigned neighborhood and the central hospital in each neighborhood is circled.
